# Classification Prediction of Jujube Variety Based on Hyperspectral Imaging: A Comparative Study of Intelligent Optimization Algorithms

**DOI:** 10.3390/foods14142527

**Published:** 2025-07-18

**Authors:** Quancheng Liu, Jun Zhou, Zhaoyi Wu, Didi Ma, Yuxuan Ma, Shuxiang Fan, Lei Yan

**Affiliations:** 1 School of Technology, Beijing Forestry University, Beijing 100083, China; liuqc@bjfu.edu.cn (Q.L.); wojiaozhoujun198@163.com (J.Z.); zhuixunWZY666@bjfu.edu.cn (Z.W.); Mdd2194155477@bjfu.edu.cn (D.M.); m19213026571@163.com (Y.M.); 2 Key Laboratory of National Forestry and Grassland Administration on Forestry Equipment and Automation, Beijing 100083, China

**Keywords:** dried jujubes, hyperspectral imaging, machine learning, intelligent optimization algorithms, variety classification, GWO, SVM

## Abstract

Accurate classification of jujube varieties is essential for ensuring their quality and medicinal value. Traditional methods, relying on manual detection, are inefficient and fail to meet the demands of modern production and quality control. This study integrates hyperspectral imaging with intelligent optimization algorithms—Zebra Optimization Algorithm (ZOA), Genetic Algorithm (GA), Particle Swarm Optimization (PSO), and Grey Wolf Optimization (GWO)—and a Support Vector Machine (SVM) model to classify jujube varieties. First, the Isolation Forest (IF) algorithm was employed to remove outliers from the spectral data. The data were then processed using Baseline correction, Multiplicative Scatter Correction (MSC), and Savitzky-Golay first derivative (SG1st) spectral preprocessing techniques, followed by feature enhancement with the Competitive Adaptive Reweighted Sampling (CARS) algorithm. A comparative analysis of the optimization algorithms in the SVM model revealed that SG1st preprocessing significantly boosted classification accuracy. Among the algorithms, GWO demonstrated the best global search ability and generalization performance, effectively enhancing classification accuracy. The GWO-SVM-SG1st model achieved the highest classification accuracy, with 94.641% on the prediction sets. This study showcases the potential of combining hyperspectral imaging with intelligent optimization algorithms, offering an effective solution for jujube variety classification.

## 1. Introduction

Jujube (*Zizyphus jujuba* Mill.) is the fruit of a plant in the Rhamnaceae family and the Ziziphus genus, with a cultivation history of over 4000 years, widely distributed across China and other regions [1]. As a traditional Chinese medicinal material, jujube holds a prominent position in the global market due to its pharmacological benefits—such as nourishing yin, generating body fluids, moistening the lungs, and calming the heart—and its rich nutritional content [2]. Dried jujube accounts for approximately 70% of the market share. However, due to factors like temperature, rainfall, and sunlight, different jujube varieties exhibit significant variations in sensory quality, nutritional content, and flavor, leading to confusion in the market and challenges in source identification. This issue is compounded by the physical similarity of different varieties, which makes adulteration and impersonation common, undermining consumer trust and complicating market regulation [3]. With increasing global trade and food safety concerns, the effective use of food traceability technology has become crucial for the sustainable development of the food industry [4].

The main production areas of jujube are concentrated in the Yellow River Basin and its surrounding regions in China, particularly in provinces such as Xinjiang, Shaanxi, Shanxi, Shandong, and Hebei [5]. In recent years, the Chinese government has strengthened protection measures for Geographical Indication Products (PGI), enhancing the market competitiveness of these protected products [6]. To ensure the competitiveness of jujube brands and traceability of their products, there is an urgent need for reliable methods to identify jujube varieties and their origins. The inspection based on expert judgment, which evaluates morphological and textural characteristics, is subjective, limited to inspecting only a small sample, and often does not accurately represent the quality of the entire batch. While modern techniques such as stable isotope analysis [7], chromatography [8], and Polymerase Chain Reaction (PCR) [9] have been widely applied in the differentiation of jujube varieties. Although these methods offer precision advantages, they typically require advanced laboratory equipment, complex sample pretreatment processes, and skilled operators, which limits their widespread application in routine settings [10]. Therefore, developing a fast, non-destructive, and efficient method to identify jujube varieties and their origins is critical.

Hyperspectral imaging (HSI) technology combines the advantages of traditional imaging techniques and spectral analysis, enabling the simultaneous acquisition of spectral and spatial information from target objects, thereby allowing for a comprehensive analysis of surface features [11]. Compared to traditional manual and chemical detection methods, HSI directly obtains the physicochemical properties of samples without the need for complex preprocessing, making it a powerful tool for rapid sample attribute detection. HSI technology provides continuous imaging across a wide range of spectral wavelengths, yielding rich spectral data that helps accurately identify different features and categories of objects [12]. In recent years, the application of HSI technology in the agricultural and food industries has grown steadily. For example, Chen, B. et al. [13] combined HSI with machine learning methods to classify pecan seed varieties. By preprocessing spectral data and performing feature extraction, the study used a support vector machine (SVM) model to classify 19 varieties of pecan seeds, achieving a classification accuracy of 96.5%. Similarly, Gong, J. et al. [14] applied HSI and partial least squares discriminant analysis (PLS-DA) to differentiate rapeseed varieties and assess seed purity, successfully identifying pure seeds even when adulterated with other varieties.

The integration of HSI with chemometrics further enhances the accuracy of jujube quality analysis, providing precise quantitative and qualitative assessments, making it a valuable tool for food safety and quality control [15]. Moreover, intelligent optimization algorithms have shown significant promise in pattern recognition and data analysis. Support Vector Machine (SVM), a widely used algorithm for classification and regression, excels at handling high-dimensional datasets and small sample sizes due to its excellent generalization ability [16]. Liu, Q. et al. [17] combined HSI to differentiate dried jujube varieties at different maturation stages, using Area Normalization (AN), Competitive Adaptive Reweighted Sampling (CARS), and Uninformative Variable Elimination (UVE) for feature selection. This approach achieved a classification accuracy of 93.1% using the SVM model. Optimizing the SVM classifier with intelligent algorithms like Particle Swarm Optimization (PSO) and Genetic Algorithm (GA) can further improve classification performance and prediction accuracy [18]. Zhang, S. et al. [19] optimized SVM parameters using the PSO algorithm to detect five types of wheat flour, achieving 100% accuracy in both the calibration and validation sets.

The objectives of this study are as follows: (1) To collect spectral data of 15 jujube samples from major production areas in China within the wavelength range of 400–1000 nm; (2) To remove outlier samples using the Isolation Forest algorithm, and apply preprocessing methods such as Multi-Scatter Correction (MSC), Baseline Correction, and the first-order Savitzky-Golay derivative (SG1st), along with dimensionality reduction and visualization techniques like Principal Component Analysis (PCA), t-Distributed Stochastic Neighbor Embedding (t-SNE), and Uniform Manifold Approximation and Projection (UMAP) for preliminary data analysis; (3) To extract characteristic wavelengths from the original and preprocessed spectra using the Competitive Adaptive Reweighted Sampling (CARS) algorithm, and build intelligent optimization algorithms, including Particle Swarm Optimization (PSO), Genetic Algorithm (GA), Zebra Optimization Algorithm (ZOA), and Grey Wolf Optimization (GWO), in conjunction with the SVM algorithm for jujube variety classification, ultimately obtaining classification results for the 15 jujube varieties.

## 2. Materials and Methods

### 2.1. Jujube Samples

**Figure 1:**
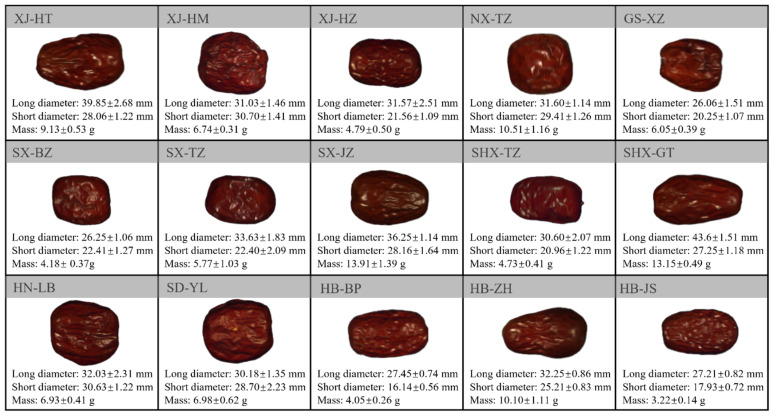
RGB Images and Statistical Data of 15 Jujube Varieties from Various Regions in China.

A total of 15 jujube varieties were collected from major jujube-producing regions across China. Specifically, *Ziziphus jujuba* cv. Hetianzao, Hamizao, and Huizao were sourced from the Xinjiang Uygur Autonomous Region; *Ziziphus jujuba* cv. Hetianzao from the Ningxia Hui Autonomous Region; *Ziziphus jujuba* cv. Xiaozao from Gansu Province; *Ziziphus jujuba* cv. Banzao, Tanzao, and Junzao from Shanxi Province; another *Ziziphus jujuba* cv. Tanzao and Goutouzao from Shaanxi Province; *Ziziphus jujuba* cv. Fupingzao, Jinsizao, and Zanghuangzao from Hebei Province; *Ziziphus jujuba* cv. Yuanlingzao from Shandong Province; and *Ziziphus jujuba* cv. Lingbaozao from Henan Province. These varieties are designated as XJ−HT, XJ−HM, XJ−HZ, NX−TZ, GS−XZ, SX−BZ, SX−TZ, SX−JZ, SHX−TZ, SHX−GT, HB−FP, HB−JS, HB−ZH, SD−YL, and HN−LB, respectively. For each variety, 180 individual samples were selected. The statistical data, including RGB images, single fruit weight, long diameter, and short diameter, for each of the different jujube varieties are summarized in Figure 1.

### 2.2. Hyperspectral Imaging System and Data Acquisition

The hyperspectral imaging (HSI) system used in this study consists of an SOC710VP hyperspectral imager (Surface Optics Co., Ltd., San Diego, CA, USA), a light source, and a computer. The light source includes two 150 W SLS CL-150 fiber-optic halogen lamps (Technquip, Pleasanton, CA, USA), positioned at a 45-degree angle to the sample surface with a lamp height of 300 mm. The system is mounted in an external frame made of 40 mm × 40 mm aluminum profiles, with overall dimensions of 1000 mm × 1000 mm × 1400 mm. To minimize interference from external ambient light, the frame is covered with a specialized light-shielding fabric to ensure 100% light blockage.

**Figure 2:**
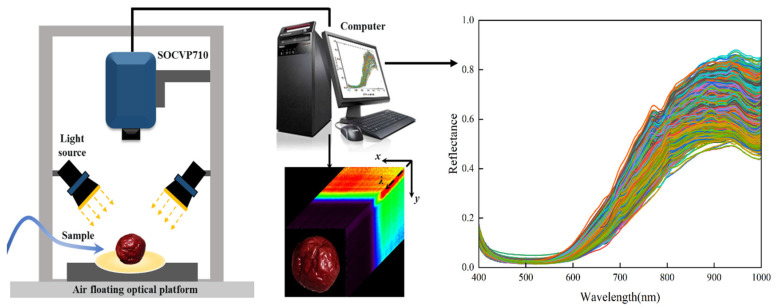
Hyperspectral Imaging System and Spectral Data Acquisition of Jujubes.

Before the experiment, the system was preheated for 30 min to optimize spectral image acquisition within the wavelength range of 400–1000 nm. During the spectral data extraction process, the hyperspectral image was first calibrated, and then the spectral data of the jujube samples were extracted. The data acquisition and extraction process is shown in Figure 2. Using threshold segmentation on the grayscale image, the jujube samples were effectively separated from the background. Following the approach outlined by Liu et al. [20], the spectral data for each jujube sample were extracted from the selected Region of Interest (ROI), consisting of 229 spectral bands. The average reflectance within each jujube sample’s ROI was then calculated to obtain the spectral data.

To eliminate the influence of external factors and instruments, it is necessary to correct the original hyperspectral image with white and dark reference images before extracting the spectrum. The correction formula can be represented as follows:

(1) $$R=\frac{R_{e}-R_{d}}{R_{w}-R_{d}}$$

where R is the correct hyperspectral image in units of relative reflectance (%); $R_{e}$ represents the reproduced original hyperspectral image; and $R_{d}$ is the black reference image obtained by turning off the light to block the camera lens. $R_{w}$ is the white reference image obtained from a 99% reflectance white plate.

### 2.3. Data Processing

#### 2.3.1. Isolation Forest (IF) algorithm

During the experiment, factors such as instrument errors, equipment malfunctions, and environmental changes may lead to the generation of abnormal samples, potentially compromising the reliability of the analysis results [21]. To address this, the study utilized the Isolation Forest (IF) algorithm, an efficient unsupervised anomaly detection method [22]. The IF algorithm assumes that anomalies are rare and significantly different from normal data in terms of attribute values, making them easier to isolate through random partitioning. Unlike traditional anomaly detection methods, IF constructs a framework around the anomaly points and recursively partitions the data space, isolating abnormal samples into leaf nodes [23]. As a non-parametric ensemble method, IF is fast, efficient, and does not require hyperparameter tuning, making it particularly suitable for unsupervised anomaly detection tasks.

#### 2.3.2. Spectral Data Preprocessing

Spectral preprocessing methods are essential for removing irrelevant information and improving the accuracy and interpretability of the model [24]. In this study, several preprocessing techniques were applied to the spectral data. First, baseline offset correction (Baseline) was performed using a linear correction model [25]. Using 400 nm and 1000 nm as ideal baseline points, the skewed baseline was adjusted to a horizontal one, enhancing sensitivity and accuracy in substance detection. To address both additive and multiplicative effects in the spectral data, Multi-Scatter Correction (MSC) was applied to reduce deviations in effective path length and scattering effects [26]. Additionally, the first-order derivative was utilized to further correct the baseline and improve the spectral resolution.

#### 2.3.3. Characteristic Variable Selection Based on CARS

Feature selection plays a crucial role in hyperspectral data analysis by extracting the most representative subset of features, which helps eliminate irrelevant ones and clarifies the relationship between data attributes and the target variable. In this study, the Competitive Adaptive Reweighted Sampling (CARS) algorithm was employed for feature band extraction [27]. CARS combines Monte Carlo sampling with Partial Least Squares (PLS) regression coefficients. The algorithm iteratively selects the most representative feature points with large absolute regression coefficients from the PLS model through adaptive weighted sampling, generating a new feature subset and performing iterative calculations. Finally, the optimal feature bands are selected through cross-validation. The CARS algorithm employs 10-fold cross-validation and 500 Monte Carlo sampling iterations, combined with the PLS regression model for feature selection. The optimal number of PLS components is determined by calculating the minimum Root Mean Square Error of Cross-Validation (RMSECV).

### 2.4. Principle and Implementation of the Algorithm

#### 2.4.1. Support Vector Machine (SVM)

This study uses the Support Vector Machine (SVM) model for classification. SVM is a supervised learning method that constructs a decision hyperplane to separate data classes optimally. By mapping data to a high-dimensional space and using a kernel function, SVM enables nonlinear classification in the original space. The goal is to find a hyperplane that maximizes the margin between classes. The Radial Basis Function (RBF) is selected as the kernel function for its effectiveness in handling non-linear relationships [28]. SVM performance depends on two key parameters: the penalty coefficient (C), which controls model complexity and prevents overfitting, and the kernel parameter (g), which adjusts the model’s ability to fit nonlinear data. Proper tuning of these parameters is essential for maximizing classification accuracy.

#### 2.4.2. Dimensionality Reduction and Visualization Analysis

Principal Component Analysis (PCA) minimizes information loss by projecting the original features onto new dimensions (principal components) with maximum variance [29]. As an unsupervised learning method, PCA calculates the eigenvalues and eigenvectors of the covariance matrix to extract principal components. The eigenvectors indicate the directions of maximum variance, while the eigenvalues represent the magnitude of that variance. The principal components are orthogonal, ensuring uncorrelated new dimensions. By projecting the data onto these components, PCA effectively reduces dimensionality while retaining key structural information.

t-Distributed Stochastic Neighbor Embedding (t-SNE) reduces dimensionality by converting Euclidean distances in high-dimensional space into conditional probabilities of similarity [30]. In high-dimensional space, similarity is measured using a Gaussian distribution, while in low-dimensional space, t-SNE uses a t-distribution with heavy tails. t-SNE minimizes the Kullback-Leibler (*KL*) divergence between the probability distributions in both spaces, ensuring that similar data points remain clustered in the low-dimensional space, preserving local structure. The mathematical expression for *KL* divergence is:

(2) $$KL\left(P\left\|Q\right)=\sum_{i}P(i\right)\log\frac{P_{\left(i\right)}}{Q\left(i\right)}$$

where, *P* and *Q* represent the probability distributions in high-dimensional and low-dimensional spaces, respectively.

Uniform Manifold Approximation and Projection (UMAP) is based on manifold learning and topological data analysis theories [31]. It starts by constructing a weighted graph to represent local relationships between data points, with edge weights determined by fuzzy simplicial sets. UMAP aims to find a low-dimensional embedding by minimizing the cross-entropy loss between the high-dimensional and low-dimensional representations, preserving both global and local data structure. The optimization objective for UMAP is:

(3) $$L=\sum_{i,j}\left[P_{ij}\log\frac{P_{ij}}{Q_{ij}}\right]$$

where, $P_{ij}$ is the similarity in high-dimensional space, and $Q_{ij}$ is the similarity in low-dimensional space.

#### 2.4.3. Zebra Optimization Algorithm (ZOA)

The Zebra Optimization Algorithm (ZOA) is an optimization algorithm inspired by the behavior of zebras in their natural environment, proposed in 2022 [32]. ZOA guides the search process by simulating the foraging and defense strategies of zebras to solve complex optimization problems. Each zebra in the algorithm represents a potential solution to the problem, and its habitat area is mapped to the search space of the problem. The position of each zebra in this space corresponds to the values of the decision variables, and the entire population of zebras can be represented in a matrix form.

(4) $$X={\left[\begin{array}{c} \begin{array}{c} X_{1}\\ \vdots \\ X_{i} \end{array}\\ \vdots \\ X_{N} \end{array}\right]}_{N\times m}={\left[\begin{array}{c} x_{1,1}\\ \vdots \\ \begin{array}{c} x_{i,1}\\ \vdots \\ x_{N,1} \end{array} \end{array}\begin{array}{c} \ldots \\ \ddots \\ \begin{array}{c} \ldots \\ ⋰\\ \ldots \end{array} \end{array}\begin{array}{c} x_{1,j}\\ \vdots \\ \begin{array}{c} x_{i,j}\\ \vdots \\ x_{N,j} \end{array} \end{array}\begin{array}{c} \ldots \\ ⋰\\ \begin{array}{c} \ldots \\ \ddots \\ \ldots \end{array} \end{array}\begin{array}{c} x_{1,m}\\ \vdots \\ \begin{array}{c} x_{i,m}\\ \vdots \\ x_{N,m} \end{array} \end{array}\right]}_{N\times m}$$

(5) $$F={\left[\begin{array}{c} \begin{array}{c} F_{1}\\ \vdots \\ F_{i} \end{array}\\ \vdots \\ F_{N} \end{array}\right]}_{N\times m}={\left[\begin{array}{c} \begin{array}{c} F(X_{1})\\ \vdots \\ F(F_{i}) \end{array}\\ \vdots \\ F(F_{N}) \end{array}\right]}_{N\times m}$$

where, X represents the number of zebras, $X_{i}$ denotes a single zebra, N is the population size of zebras, and m is the number of problem variables. The value $x_{i,j}$ represents the value of the *j*-th decision variable for the *i*-th zebra. The goal for each zebra is to adjust its position continually to find the optimal solution.

During each iteration, the ZOA algorithm updates the population’s positions by simulating two key behaviors of zebras: foraging and defense strategies, representing exploration and protection of the search space, respectively.

Foraging Phase: Zebras update their positions based on the leader zebra’s location, which represents the best solution in the current population. This phase explores the search space, guiding the population toward potential global optima.

Defense Phase: When facing a “predator,” zebras use a defense strategy, simulated as a local search. The leader zebra intensifies the search around its position to avoid being trapped in a local optimum.

#### 2.4.4. Genetic Algorithm (GA)

The Genetic Algorithm (GA), proposed by John Holland in the 1970s, is an optimization method that simulates biological evolution [33]. Based on Darwin’s theory of natural selection and genetic inheritance, GA searches for the global optimal solution by mimicking the evolutionary processes of crossover, mutation, and selection in biological populations. The selection of the best individuals in the population is based on their fitness, with fitter individuals having a higher chance of reproduction. A common selection strategy is the roulette wheel method, where the probability of selection is proportional to an individual’s fitness. The selection probability can be expressed as:

(6) $$P(x_{i})=\frac{f(x_{i})}{\sum_{i=1}^{N}f(x_{i})}$$

where, $P(x_{i})$ is the probability of individual $x_{i}$ being selected, $f(x_{i})$ is the fitness value of individual $x_{i}$, and *N* is the population size.

The crossover operation simulates genetic recombination by crossing the genes of two parent individuals to generate new offspring. The purpose of crossover is to explore new solutions in the solution space through genetic recombination. The crossover point is chosen randomly, and the crossover operation can be expressed as:

(7) $$x_{i}^{\prime }=\alpha x_{i1}+(1-\alpha )x_{i2}$$

where, $x_{i1}$ and $x_{i2}$ are the parent individuals, $x_{i}^{\prime }$ is the new individual, and *α* is the crossover coefficient, which ranges from [0, 1].

The mutation operation randomly alters an individual’s genes, increasing population diversity and preventing the algorithm from getting stuck in a local optimum. The mutation operation can be expressed as:

(8) $$x_{i}^{mutated}=x_{i}+\Delta_{x}$$

where, $\Delta_{x}$ represents the mutation variable, which is typically a small random perturbation.

#### 2.4.5. Particle Swarm Optimization (PSO)

The Particle Swarm Optimization (PSO) algorithm is a global optimization technique based on swarm intelligence [34]. It simulates the flying behavior of particles within the solution space to find the optimal solution. Initially, a set of randomly generated particles represent potential solutions. Each particle adjusts its position and velocity in subsequent iterations, gradually converging to the global optimum.

The particle swarm consists of *N* particles, each of which is represented as a *D* dimensional vector. The dimension *D* corresponds to the number of design variables. The position of a particle in the space can be represented as:

(9) $$x_{i}=\left[x_{i1},x_{i2},\ldots ,x_{iD}\right]$$

where, $x_{i}$ denotes the position of the *i*-th particle in the *D*-dimensional design space. $x_{ij}$ represents the value of the j-th design variable for the *i*-th particle. The spatial position of particles is a solution in the objective optimization problem. The fitness of particles can be evaluated through a fitness function, which allows the quality of particles to be measured based on the fitness value.

The flying velocity of a particle is also a *D* dimensional vector, represented as:

(10) $$v_{i}=\left[v_{i1},v_{i2},\ldots ,v_{iD}\right]$$

where, $v_{i}$ is the flying velocity of the *i*-th particle, which determines the direction and speed of the particle’s position update in each iteration. The velocity update rule for the particle is adjusted based on its current position, its personal best position, and the global best position of all particles.

(11) $$v_{ij}\left(t+1\right)=\omega v_{ij}\left(t\right)+c_{1}r_{1}\left(p_{ij}-x_{ij}\left(t\right)\right)+c_{2}r_{2}\left(g_{j}-x_{ij}\left(t\right)\right)$$

(12) $$x_{ij}\left(t+1\right)=x_{ij}\left(t\right)+v_{ij}\left(t+1\right)$$

where, $v_{ij}\left(t\right)$ and $x_{ij}\left(t\right)$ are the velocity and position of particle i in the *j*-th dimension at time *t*, $p_{ij}$ is the historical best position of particle i in the *j*-th dimension, $g_{j}$ is the global best position of all particles in the *j*-th dimension, $r_{1}$ and $r_{2}$ are random numbers between [0, 1], $c_{1}$ and $c_{2}$ are acceleration constants, and *ω* is the inertia weight, which controls the influence of the particle’s previous velocity.

#### 2.4.6. Grey Wolf Optimizer (GWO)

The GWO employs a simulation of gray wolf hunting behavior to identify the optimal solution [35]. A strict hierarchy exists within a wolf pack, with the alpha wolf occupying the highest rank and serving as the pack leader. Subsequently, the *β*-wolf, *δ*-wolf, and *ω*-wolf are in descending order of status in the pack. Thus, the solution represented by *α*-wolf is considered the optimal solution, while the solutions represented by *β*-wolf, *δ*-wolf, and *ω*-wolf are the second-best, third-best, and residual solutions, in that order.

Gray wolves surround their prey while hunting, and this behavior can be mathematically represented as follows.

(13) $$\vec{D}=\left|\vec{C}\cdot \vec{X_{p}}\left(t\right)-\vec{X}\left(t\right)\right|$$

(14) $$\vec{X}\left(t+1\right)=\vec{X_{p}}\left(t\right)-\vec{A\cdot }\vec{D}$$

where, $\vec{X_{p}}\left(t\right)$ denotes the location of the prey, $\vec{X}\left(t\right)$ denotes the location of the gray wolf, the distance vector between the prey and the gray wolf is depicted by $\vec{D}$, $\vec{A}$ and $\vec{D}$ are the coefficient vectors, which is given by the following formulae:

(15) $$\vec{A}=\left|2\cdot \vec{a}\cdot \vec{\lambda_{1}}-\vec{a}\right|$$

(16) $$\vec{C}=2\cdot \vec{\lambda_{2}}$$

where the value of $\vec{a}$ declines in a linear fashion between 2 and 0, while the vectors $\vec{\lambda_{1}}$ and $\vec{\lambda_{2}}$ are randomly generated over the interval [0, 1]. $\vec{C}$ acts as a random weight factor, the value of $\vec{C}$ can be a random number greater than 1 or less than 1. It determines whether the distance information in each direction is emphasized or weakened when calculating the prey position, which helps to find the global optimal solution.

It has been demonstrated that wolves are able to recognize the location of their prey and update their positions accordingly. In this example, the positions indicated by α, β and δ wolves represent the first three optimal solutions and collectively serve to direct the collective speculation of the pack regarding the optimal prey location. In contrast, the positions indicated by ω wolves are updated based on the most optimal search position.

(17) $$\vec{D_{a}}=\left|\vec{C_{1}}\cdot \vec{X_{a}}-\vec{X}\right|,\;\vec{X_{1}}=\vec{X_{a}}-\vec{A_{1}\cdot }(\vec{D_{a})}$$

(18) $$\vec{D_{\beta }}=\left|\vec{C_{2}}\cdot \vec{X_{\beta }}-\vec{X}\right|,\;\vec{X_{2}}=\vec{X_{\beta }}-\vec{A_{2}\cdot }(\vec{D_{\beta })}$$

(19) $$\vec{D_{\delta }}=\left|\vec{C_{3}}\cdot \vec{X_{\delta }}-\vec{X}\right|,\;\vec{X_{3}}=\vec{X_{\delta }}-\vec{A_{3}\cdot }(\vec{D_{\delta })}$$

(20) $$\vec{X}\left(t+1\right)=\left(\vec{X_{1}}+\vec{X_{2}}+\vec{X_{3}}\right)/3$$

where $\vec{D_{a}}$, $\vec{D_{\beta }}$ and $\vec{D_{\delta }}$ are the distance vectors of *ω*-wolf concerning *a*-wolf, *β*-wolf and δ-wolf, $\vec{X_{a}}$, $\vec{X_{\beta }}$ and $\vec{X_{\delta }}$ are the positions of *a*-wolf, *β*-wolf and *δ*-wolf.

Gray wolves attack prey when it stops moving. Mathematically, this can be described as follows: as the value of $\vec{a}$ gradually decreases from 2 to 0 during iterations, the value of $\vec{A}$ decreases accordingly. Once the value of $\vec{A}$ enters the interval [−1, 1], the gray wolf attacks its prey. As the algorithm iterates, the wolves continue to adjust their search strategy, ultimately achieving the capture of the prey. At this juncture, the location of the prey represents the optimal solution sought by the algorithm.

### 2.5. Sample Splitting and Optimizer Parameter Settings

In this study, spectral data for 15 jujube varieties were collected. To avoid data redundancy and overfitting, the dataset with outliers removed was split into a calibration set and a prediction set in a 3:1 ratio using the Kennard-Stone (KS) algorithm [36]. Four intelligent optimization algorithms (ZOA, GA, PSO, GWO) were used to optimize the hyperparameters (C and g) of the SVM to enhance classification accuracy. A fitness function was defined for each algorithm, with the search conducted within a parameter space. The parameters included a population size of 15, a maximum iteration count of 30, a parameter dimension of 2, and lower and upper bounds of [0.001, 0.001] and [100, 100], respectively. The performance of each optimization algorithm was evaluated via its fitness curve and classification accuracy. To ensure unbiased evaluation, the preprocessing was applied only after splitting the data into calibration and prediction sets. Additionally, the classification performance of each algorithm was analyzed using confusion matrices on both the calibration and prediction sets. The SVM models based on different optimization algorithms were compared for accuracy and classification effectiveness, highlighting the strengths and limitations of each method for high-dimensional dataset classification tasks.

## 3. Results

### 3.1. Removal of Abnormal Data

**Figure 3:**
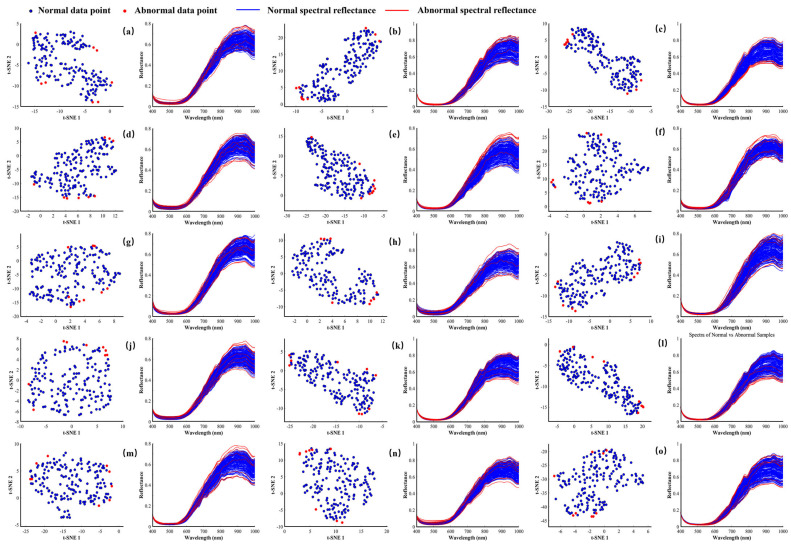
Removal of Outlier Samples Based on the Isolation Forest Algorithm. (**a**–**o**) represent jujube samples from different classes, in the following order: XJ−HT, XJ−HM, XJ−HZ, NX−TZ, GS−XZ, SX−BZ, SX−TZ, SX−JZ, SHX−TZ, SHX−GT, HN−LB, SD−YL, HB−FP, HB−ZH, and HB−JS.

Figure 3 shows the effect of outlier removal on the spectral data of 15 jujube varieties using the Isolation Forest (IF) algorithm. The algorithm calculates the anomaly score of each data point by averaging its path length across all isolation trees to identify outliers. Visual analysis of the figure reveals a clear distinction between normal data points (blue) and outliers (red) in the reduced-dimensional space. Outliers are scattered on the plot’s periphery, while normal data points cluster in the center. This separation confirms that the Isolation Forest algorithm effectively identifies and removes outliers.

Furthermore, a comparison of the spectral reflectance curves reveals that outlier data generally exhibit significant deviations and erratic fluctuations, appearing at the upper and lower extremes of the curve, lacking consistency. In contrast, the spectral curves of normal samples follow a consistent, orderly trend in the middle range of the curve. This pattern further confirms the effectiveness of the Isolation Forest algorithm in identifying and removing anomalous samples. A total of 2565 jujube samples were obtained. By accurately removing outliers, the Isolation Forest algorithm improved data quality and provided more reliable input for subsequent model training, minimizing the interference of outlier data and significantly enhancing the accuracy and robustness of the classification model.

### 3.2. Spectral Characteristics

**Figure 4:**
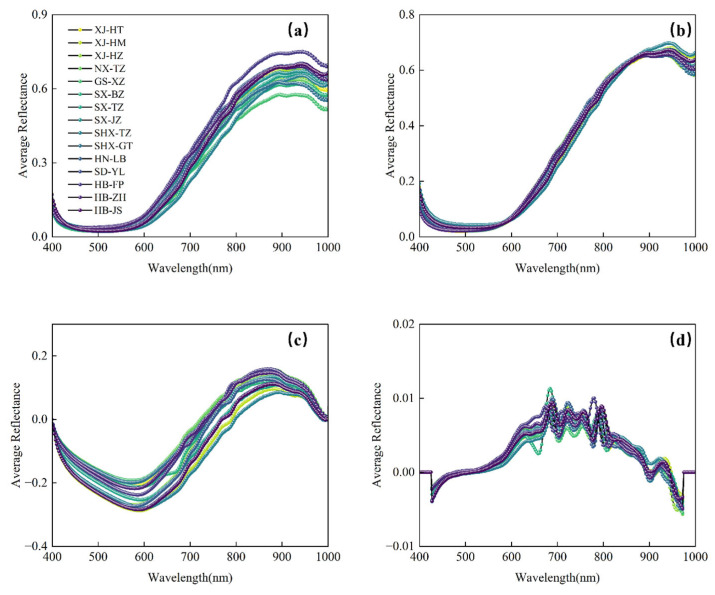
Average spectral curves of raw and different preprocessing methods. (**a**) Raw spectral; (**b**) MSC spectral; (**c**) Baseline spectral; (**d**) SG1st spectral.

After removing outliers, the average reflectance spectra of 15 jujube samples were constructed, as shown in Figure 4. Panel 4a displays the spectral reflectance curves of different jujube varieties. While the spectral curves exhibit a consistent trend, notable differences in reflectance values are observed, particularly in the 600–700 nm range [37]. These differences indicate that spectral features can distinguish the quality of jujubes at different stages. Small peaks and valleys at 650 and 670 nm correspond to chlorophyll absorption characteristics, primarily due to C-H stretching vibrations. The broad peak around 870 nm is linked to the O-H bending vibration of water, while the absorption dips around 920 and 970 nm reflect the O-H bond’s third harmonic absorption in water molecules [38]. Analyzing these characteristic bands provides valuable insights into the spectral features of jujube samples, aiding in quality analysis.

To further eliminate the impact of outlier data, this study performed Baseline, MSC, and SG1st preprocessing on the spectral data. These preprocessing methods removed the interference caused by spectral data noise, baseline drift, and scattering effects, thus improving the accuracy and reliability of the subsequent analysis, as shown in Figure 4b–d.

### 3.3. PCA, t-SNE, and UMAP Visualization Analysis

**Figure 5:**
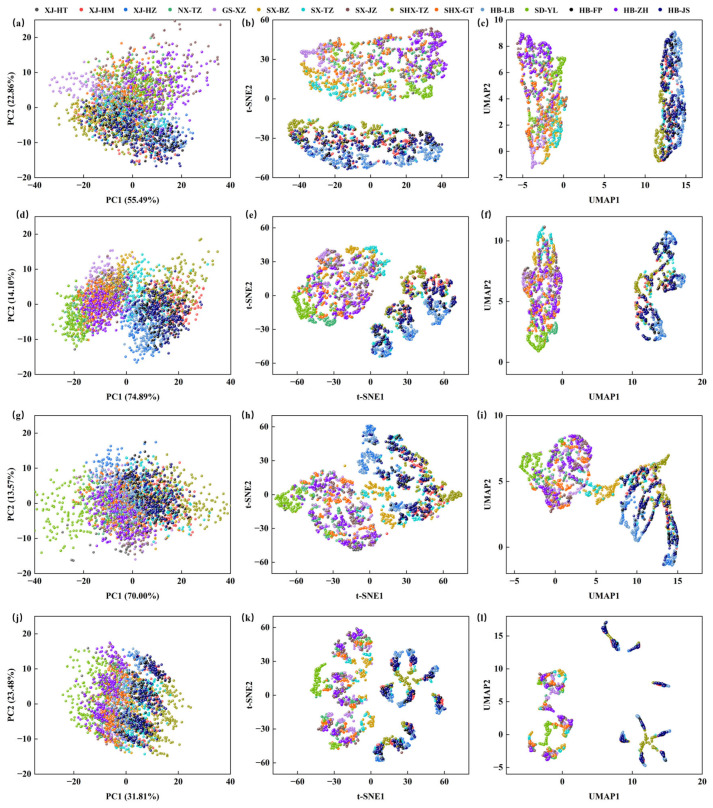
PCA, t-SNE, and UMAP Visualization Distribution of Spectral Data. (**a**) Raw-PCA; (**b**) Raw-t-SNE; (**c**) Raw-UMAP; (**d**) Baseline-PCA; (**e**) Baseline -t-SNE; (**f**) Baseline -UMAP; (**g**) MSC-PCA; (**h**) MSC-t-SNE; (**i**) MSC-UMAP; (**j**) SG1st-PCA; (**k**) SG1st -t-SNE; (**l**) SG1st -UMAP.

A comparative analysis of PCA, t-SNE, and UMAP dimensionality reduction results, using both the original and preprocessed spectral data (Baseline, MSC, SG1st), as shown in Figure 5. The analysis revealed that as preprocessing steps were applied, the separation between samples increased, leading to significantly improved clustering. In the original data visualizations, there was considerable overlap, indicating noise and redundancy. After Baseline preprocessing, reflectance intensity was corrected, enhancing sample distribution, especially in t-SNE and UMAP, where the distinction between normal and outlier samples became clearer. Further application of MSC and SG1st preprocessing methods optimized clustering in the reduced space. MSC removed scattering effects and improved resolution, while SG1st reduced high-frequency noise, making spectral features more prominent for better clustering and visualization in t-SNE and UMAP.

Overall, t-SNE and UMAP demonstrated superior sample separation capabilities compared to PCA when handling high-dimensional data. However, despite the increased differentiation between samples in the reduced space, the discrimination effect was still limited, with some overlap remaining between similar varieties. t-SNE is particularly effective at preserving local structure and visualizing clusters, but it does not preserve global relationships and cannot be used for projecting or classifying new data points. UMAP builds upon t-SNE by retaining more global structure, making it suitable for both visualization and dimensionality reduction tasks, though it does not provide insights into feature importance. PCA, a linear technique, preserves global structure and offers feature importance through component loadings, but its ability to capture non-linear relationships in the data is limited.

Therefore, to further improve classification accuracy, it is recommended to construct more precise classification models using supervised learning methods such as SVM. Specifically, four intelligent optimization algorithms (ZOA, GA, PSO, GWO) were applied to optimize the SVM hyperparameters (C and g), which will further enhance the model’s accuracy and generalization ability. By optimizing these two key hyperparameters, the flexibility of the classification boundary and the influence range of the kernel function can be effectively adjusted, improving the SVM’s performance on complex datasets.

### 3.4. Results of CARS Feature Wavelength Selection

**Figure 6:**
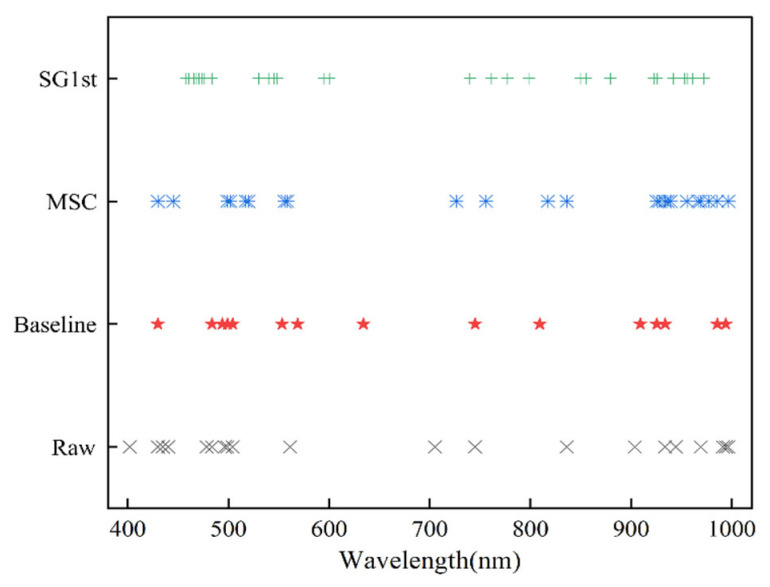
Range of Characteristic Variables in Raw and preprocessing Spectral Selection.

Feature extraction aims to identify key information from raw and preprocessed spectral data, reducing processing time, simplifying model calibration, and improving model robustness. In this study, the CARS algorithm was used to select wavelengths from raw spectral data and data preprocessed with Baseline, MSC, and SG1st, aiming to extract the most representative features. The distribution of these feature variables is shown in Figure 6. In the CARS-RAW results, 20 feature variables were selected, accounting for 8.73% of the total original spectral data. This indicates that, despite the raw data containing redundancy and noise, CARS effectively filtered out key wavelengths, significantly reducing the dimensionality. In the CARS-Baseline, CARS-MSC, and CARS-SG1st results, 14, 16, and 28 feature variables were selected, representing 6.11%, 6.98%, and 12.23% of the total data, respectively. These results suggest that different preprocessing methods influenced feature selection, with SG1st preprocessing showing a significant increase in the number of selected features due to its smoothing and denoising effects.

### 3.5. Results of Intelligent Optimization Algorithm Classification

#### 3.5.1. Fitness Curve Analysis of Intelligent Optimization Algorithms

**Figure 7:**
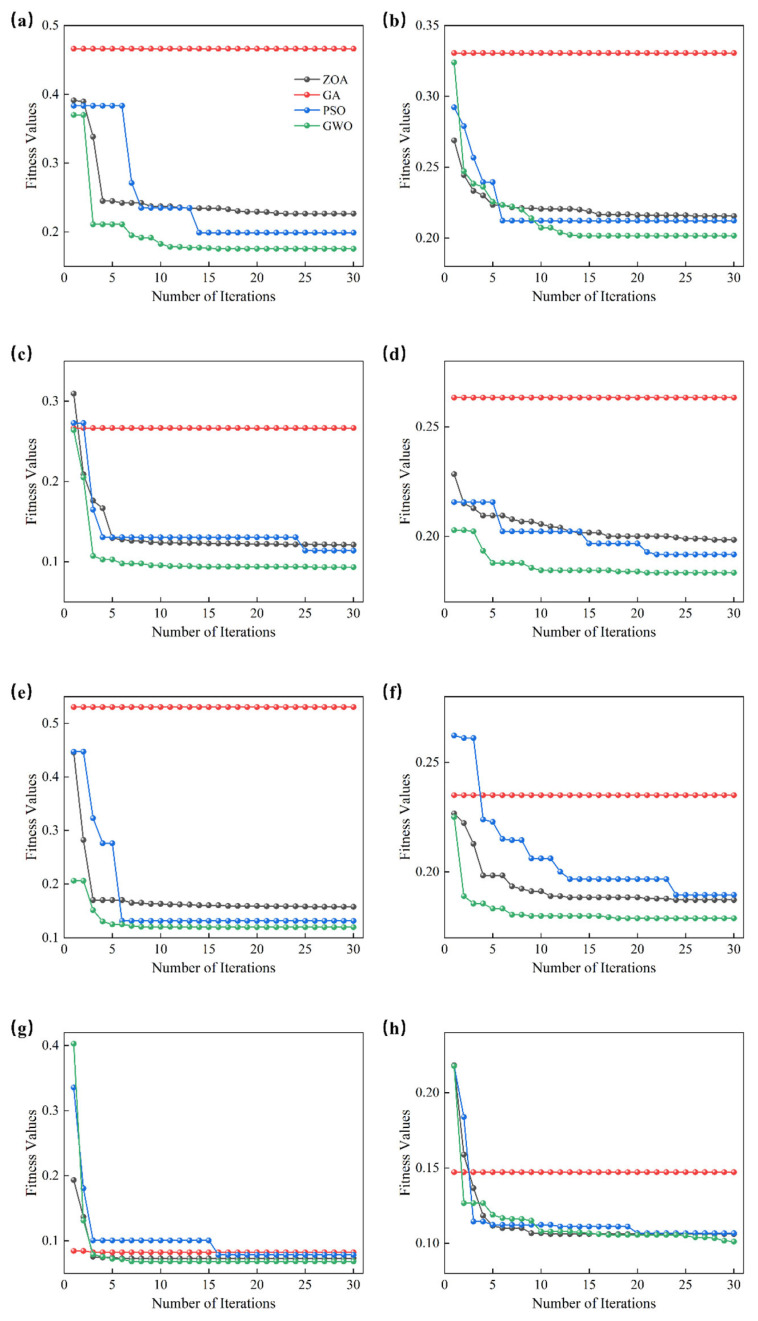
Fitness Curves Based on Different Intelligent Optimization Algorithms. (**a**) RAW; (**b**) RAW-CARS; (**c**) Baseline; (**d**) Baseline-CARS; (**e**) MSC; (**f**) MSC-CARS; (**g**) SG1st; (**h**) SG1st-CARS.

Figure 7 displays the fitness curves of four intelligent optimization algorithms (ZOA, GA, PSO, GWO) in optimizing the SVM hyperparameters (C and g). The x-axis of each subplot represents the number of iterations, and the y-axis represents the fitness value, reflecting the changes in the classification model’s performance after each iteration.

For the raw spectral data (Figure 7a), all optimization algorithms initially show a significant decline in fitness values. Notably, the fitness curve of GA remains flat, which could be due to GA getting trapped in a local optimum during the search process with the same parameter settings, preventing further optimization and halting the decrease in fitness value. In contrast, PSO, GWO, and ZOA show a rapid decline followed by a gradual fluctuation and eventual stabilization, indicating good optimization performance. Specifically, the GWO algorithm performs the best, with its fitness value continuously decreasing throughout the iterations and eventually stabilizing at the lowest fitness value, demonstrating GWO’s strong ability in global search and parameter optimization. While GA shows strong convergence in the early iterations, its final fitness value is relatively high, possibly due to premature convergence. ZOA, though not as fast as GWO and PSO in terms of convergence and final result, still exhibits strong optimization ability. PSO demonstrates a stable downward trend in fitness values throughout the optimization process, eventually achieving results comparable to GWO.

Figure 7b shows the optimization results after performing CARS feature selection on the original spectral data. By comparing the fitness curves in Figure 7a,b, it is evident that CARS feature selection significantly optimized the data structure, leading to improved convergence speed for all optimization algorithms on the feature-selected data. Notably, after CARS preprocessing, the fitness value decline speed of GWO and ZOA was noticeably faster, and they converged to a lower fitness value, highlighting the significant effect of CARS in data dimensionality reduction and noise suppression. However, it is worth noting that after CARS preprocessing, the stable fitness value of GWO approaches the result from the raw spectral data, indicating that the CARS algorithm plays a crucial role in improving the classification performance of high-dimensional datasets and optimizing the process.

GA operates through “selection, crossover, and mutation” to explore the search space, but when the population lacks diversity, it is prone to premature convergence to local optima, especially in complex high-dimensional problems. In the optimization of SVM hyperparameters, as shown in Figure 7, GA’s fitness curve levels off with a relatively high final fitness value, indicating early convergence and failure to further optimize the solution. A comparative analysis reveals that different preprocessing methods had a significant impact on the optimization process. The optimization effect for the raw spectral data was relatively weak, suggesting that the unprocessed spectral data contained a considerable amount of noise and redundant information. As the data was processed through preprocessing methods such as Baseline, MSC, and SG1st, the structure of the feature space was significantly improved, and the performance of the optimization algorithms was enhanced. Particularly under SG1st preprocessing, the fitness values of all optimization algorithms showed faster convergence and lower fluctuations, indicating that the SG1st method effectively removed noise and improved the resolution of the data, allowing the optimization algorithms to find the optimal solution more quickly [14]. It is worth noting that in Figure 7g, the GA curve shows relatively better convergence and the lowest fitness value, which can be primarily attributed to the introduction of the SG1 preprocessing method. SG1 enhances the resolution of the data and removes noise, effectively improving the GA’s search process. This helps prevent early convergence and allows GA to find better solutions. By combining different preprocessing methods and intelligent optimization algorithms, the accuracy and generalization ability of the SVM model in jujube variety classification can be effectively improved. The combination of CARS and SG1st preprocessing not only optimized the data quality but also reduced data redundancy and noise interference during model training, thereby enhancing the robustness of the model.

#### 3.5.2. Classification Accuracy of Optimized SVM Models with Intelligent Algorithms

**Figure 8:**
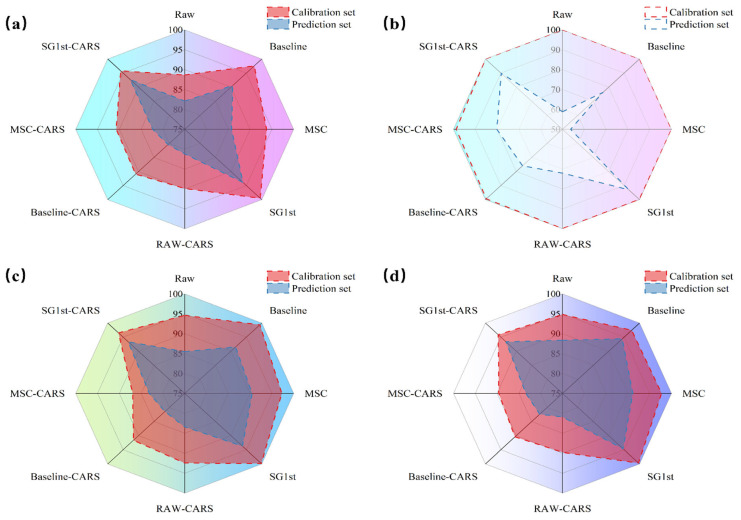
Jujube Variety Classification Results Based on Different Intelligent Optimization Algorithms. (**a**) ZOA Optimization Algorithm; (**b**) GA Optimization Algorithm; (**c**) PSO Optimization Algorithm; (**d**) GWO Optimization Algorithm.

The Figure 8 displays the classification accuracy of the calibration and prediction sets after optimizing the SVM model with four intelligent optimization algorithms (ZOA, GA, PSO, GWO). Each optimization algorithm is paired with different preprocessing methods, including SG1st, MSC, and Baseline, to investigate their impact on the model’s performance. Specifically, Figure 8a shows the classification accuracy of the ZOA-SVM model on the calibration and prediction sets after combining different preprocessing methods with CARS feature selection. The radar chart’s center represents the starting point, with the scale extending outward to represent classification accuracy, providing a clear comparison between calibration and prediction sets. Each curve in Figure 8a shows the performance under different preprocessing methods and CARS feature selection. The results indicate that the modeling results after preprocessing show varying degrees of improvement compared to the raw data.

The results demonstrate a significant improvement in classification accuracy after applying preprocessing techniques compared to the raw spectral data. Among the preprocessing methods, SG1st achieved the best prediction results, with a classification accuracy of 93.856% on the prediction set, marking an 11.765% improvement over the classification accuracy of 82.091% achieved on the raw spectral prediction set. After CARS feature selection, although the data dimensions were significantly compressed, good classification results were still obtained, with RAW-CARS achieving a classification accuracy of 81.176% on the prediction set, nearly matching the classification accuracy of the raw spectral prediction set. Notably, SG1st-CARS achieved a classification accuracy of 92.679% on the prediction set, a 10.588% improvement compared to the classification accuracy of the raw spectral prediction set.

From Figure 8b–d, it is also evident that the classification results after optimization of each classifier show that, through the analysis of the four intelligent optimization algorithms (ZOA, GA, PSO, GWO) in SVM hyperparameter optimization, the GWO-SVM model has the best overall performance. Combining the analysis in Section 3.5.1., the GWO-SVM model achieves rapid convergence with fewer iterations, avoiding overfitting, and ultimately finding the optimal solution. Similarly, the best prediction result was obtained under SG1st preprocessing, as shown in Figure 8d, where the classification accuracy reached 94.641%. Additionally, SG1st-CARS achieved a solid classification accuracy of 93.333%, further demonstrating the effectiveness of the SG1st preprocessing method in enhancing classification performance.

ZOA also demonstrated good global search capability and strong stability during optimization. Although it slightly lagged behind GWO in convergence speed and final results, it still exhibited strong optimization potential. PSO showed a stable convergence process and good stability, while GA encountered local optimum issues, failing to further optimize the solution space, resulting in a higher final fitness value and insufficient generalization ability. The optimization results after combining with CARS feature selection indicate a significant improvement in algorithm performance, particularly for GWO and ZOA. Feature selection via CARS significantly enhanced the convergence speed and stability of these algorithms, allowing for faster and more consistent optimization. The data after CARS optimization enhanced the convergence speed and stability of the algorithm, validating the crucial role of CARS in improving the classification performance of high-dimensional datasets.

### 3.6. Confusion Matrix Analysis of SG1st and SG1st-CARS Under the GWO-SVM Model

**Figure 9:**
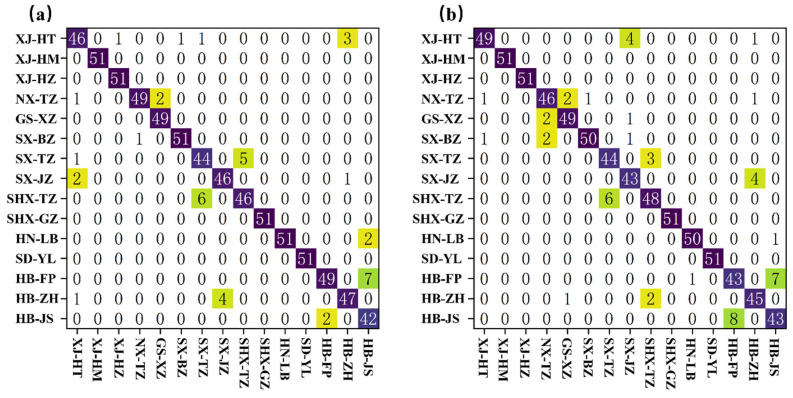
Confusion Matrix for Classification of 15 Jujube Varieties. (**a**) GWO-SVM-SG1st; (**b**) GWO-SVM-SG1st-CARS.

Figure 9a,b display the confusion matrices for SG1st and SG1st-CARS preprocessing under the GWO-SVM model, reflecting the effectiveness of these two preprocessing methods in jujube variety classification. In Figure 9a, SG1st preprocessing significantly improved the classification accuracy, with the majority of the predicted results concentrated along the diagonal, indicating that most samples were correctly classified. However, some misclassifications still occurred, such as XJ-HT (46 correct classifications) being misclassified as XJ-HM (3 misclassifications), and XJ-HZ being misclassified as NX-TZ (2 misclassifications). These errors mainly occurred between similar varieties. Additionally, misclassifications of HB-FP and HB-ZH (7 and 4 misclassifications, respectively) indicate a significant overlap in the feature space between these two varieties, making it difficult for the model to effectively distinguish them.

Figure 9b shows the results after CARS feature selection with SG1st-CARS preprocessing, which significantly reduced redundancy and noise in the spectral data and optimized the distribution of the feature space. Feature selection improved the model’s ability to distinguish between classes, especially in reducing misclassifications between XJ-HT and XJ-HM. However, XJ-HT (49 correct classifications) was still misclassified as XJ-HM (4 misclassifications), and the misclassification of HB-FP and HB-ZH remained largely unchanged. In some cases, the number of misclassifications actually increased (e.g., HB-FP had 7 misclassifications). This suggests that, although CARS feature selection optimized the data, the effect on distinguishing some similar varieties remains limited. Overall, SG1st-CARS preprocessing showed certain advantages in improving classification performance, particularly in reducing redundant information and optimizing the feature space. However, for some varieties that are difficult to differentiate, further optimization of the feature selection algorithm is still needed.

## 4. Discussion

As a traditional medicinal and food ingredient in China, accurately classifying jujube varieties is crucial for ensuring their quality and medicinal value. By leveraging spectral data and advanced optimization algorithms, this research offers an efficient approach for classifying jujube varieties, providing valuable technical support for quality assessment, traceability systems, and market strategies within the jujube industry. In this study, we selected a richer set of sample data and achieved effective differentiation of 15 types of dried jujubes from the main cultivation regions, with a classification accuracy of 94.61%. This accuracy is higher compared to that of other studies. Qi, Z. et al. [39] classified five varieties of jujubes from five regions of China (Henan, Shanxi, Xinjiang, Hebei, and Gansu) using spectral data from 900–1700 nm. Their method, based on the fuzzy improved Linear Discriminant Analysis (FiLDA) algorithm and K-Nearest Neighbors (KNN), achieved the highest classification accuracy of 94.4%. Li, X. et al. [25] conducted traceability research on jujubes from four locations: Alaer, Hotan, Ruoqiang, and Zhangye. They used near-infrared spectroscopy combined with a Convolutional Neural Network (CNN), achieving a recognition accuracy of 94.25%.

The application of intelligent optimization algorithms has proven to enhance the efficiency and accuracy of various classification tasks in diverse fields, particularly in agriculture and food quality control. Wu, X. et al. [40] used near-infrared spectroscopy combined with the Adaboost- common vectors linear discriminant analysis (CLDA) technique to classify red jujube samples from different cultivation areas and varieties. The results showed that Adaboost-CLDA performed excellently across various K values, especially when K = 7, where the classification accuracy was close to 100%. Cui, T. et al. [41] employed four classification models—Orthogonal Partial Least Squares Discriminant Analysis (OPLS-DA), Random Forest (RF), GWO-SVM, and Convolutional Neural Network (CNN)—to distinguish between Fructus Gardeniae Grandiflorae (FGG). Among these, GWO-SVM outperformed the other models, achieving a classification accuracy of 95.12%. The GWO-SVM method enhanced the traditional SVM through gray wolf optimization, making it more efficient in handling complex, nonlinear, and high-dimensional data. This study investigates the application of four intelligent optimization algorithms (ZOA, GA, PSO, GWO) within the Support Vector Machine (SVM) model, combined with various spectral preprocessing methods, to optimize the classification of jujube varieties. This study demonstrates the effectiveness of integrating intelligent optimization algorithms with spectral preprocessing techniques to enhance classification accuracy and improve overall model performance [42].

Future research could focus on refining feature selection methods by exploring deep learning-based feature selection algorithms or adaptive techniques to better handle complex datasets. Additionally, considering the performance variations of optimization algorithms across different datasets, it would be valuable to combine multiple optimization algorithms into an integrated framework. This approach could further enhance global search capabilities and improve classification precision.

## 5. Conclusions

This study explores optimization methods for jujube variety classification by combining four intelligent optimization algorithms (ZOA, GA, PSO, GWO) with the SVM model. The results show that the GWO algorithm performs the best in terms of global search and generalization capabilities, significantly improving classification accuracy. The SG1st preprocessing method optimized the classification performance by enhancing the spectral data resolution, while CARS feature selection further reduced data redundancy and optimized the feature space. The GWO-SVM-SG1st model achieved the best prediction result with a classification accuracy of 94.641%. The GWO-SVM-SG1st-CARS model also achieved good prediction accuracy (93.333%), but there are still some limitations in distinguishing between certain varieties. Overall, this study provides effective technical support for the efficient classification of jujube varieties and offers practical insights for future research on optimization algorithms and feature selection methods.

## Data Availability

The original contributions presented in this study are included in the article. Further inquiries can be directed to the corresponding authors.
